# Ships, ports and particulate air pollution - an analysis of recent studies

**DOI:** 10.1186/1745-6673-6-31

**Published:** 2011-12-05

**Authors:** Daniel Mueller, Stefanie Uibel, Masaya Takemura, Doris Klingelhoefer, David A Groneberg

**Affiliations:** 1 Department of Toxicology, Institute of Occupational Medicine, Social Medicine and Environmental Medicine, Goethe-University, Frankfurt, Germany

## Abstract

The duration of use is usually significantly longer for marine vessels than for roadside vehicles. Therefore, these vessels are often powered by relatively old engines which may propagate air pollution. Also, the quality of fuel used for marine vessels is usually not comparable to the quality of fuels used in the automotive sector and therefore, port areas may exhibit a high degree of air pollution. In contrast to the multitude of studies that addressed outdoor air pollution due to road traffic, only little is known about ship-related air pollution. Therefore the present article aims to summarize recent studies that address air pollution, i.e. particulate matter exposure, due to marine vessels. It can be stated that the data in this area of research is still largely limited. Especially, knowledge on the different air pollutions in different sea areas is needed.

## Introduction

**Figure 1:**
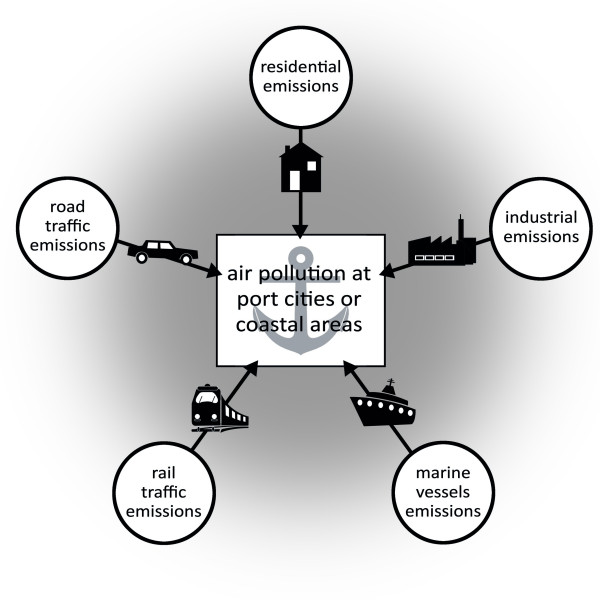
**Factors that can influence air quality in port cities and coastal areas**.

Air quality issues are extremely important for both occupational and environmental health. In this respect, numerous airborne factors negatively influence human health [1-6]. In port cities and coastal areas many sources of air pollution can be found. These air pollution sources are ship traffic, industry, rail traffic, and usual sources such as residential emissions (Figure 1). Whereas numerous studies on road traffic-related air pollution have been conducted in the past, only little is known about the magnitude and effects of air pollution due to marine vessels. According to the U.S. environmental protection agency (EPA) particulate matter (PM) is one of the six common air pollutants [7]. PM can be categorized to the main fractions such as PM10, PM2.5 and ultrafine particles (UFP). PM10 and PM2.5 are defined as particulate matter with a diameter of 10 micrometers (μm) respectively 2.5 μm collected with 50% efficiency by a sampling collection device [8]. UFP are particles with a diameter less than or equal to a nominal 0.1 μm. These small particles can depose deep in the respiratory tract, and there are multiple proven associations between these particles and acute or chronic health effects [9-20]. Therefore, this review focuses on air pollution by PM emission, especially PM10 and PM2.5 emissions, due to shipping and port activities.

## Measurements of PM concentration in coastal regions

In a multi-year study conducted from 2003 to 2007 by Pandolfi et al., ambient PM10 and PM2.5 data were collected at four sampling locations around the Bay of Algeciras in southern Spain. To identify major PM sources - with particular attention paid to the quantification of total shipping emissions - positive matrix factorization models were used [21]. After that, the impact of the emissions from the harbor of Algeciras and vessel traffic at the Western entrance of Mediterranean Sea were quantified. For the estimation of shipping dependent PM emissions, ambient levels of vanadium (V), nickel (Ni), lanthanum (La) and cerium (Ce) were used as markers in this study [21]. According to the scientists, the shipping emissions were characterized by La/Ce ratios between 0.6 and 0.8 and V/Ni ratios around 3 for both PM10 and PM2.5. It was estimated that the direct contribution from shipping in the Bay of Algeciras PM10 was 1.4 to 2.6 μg/m^3 ^(3-7%) and PM2.5 concentration was 1.2 to 2.3 μg/m^3 ^(5-10%). The study demonstrated further, that the total contribution from shipping reached 4.7 μg/m^3 ^(13%) for PM10 and 4.1 μg/m^3 ^(17%) for PM2.5 [21].

In another study Agrawal et al. investigated the impact of primary fine particulate matter PM2.5 from ship emissions within the Southern California Air Basin. In this study also V and Ni were used as marker for the shipping emissions [22]. V and Ni were measured in stack emissions of in-use ocean-going vessels (OGVs) and then compared with ambient measurements made at 10 monitoring stations throughout Southern California. The researcher could demonstrate that the transition metals V and Ni are robust markers for the combustion of heavy fuel oil in OGVs [22]. In this respect, it was found that ambient measurements of fine particulate V and Ni within Southern California are shown to decrease inversely with increased distance from the ports of Los Angeles and Long Beach. To estimate the primary PM2.5 contributions from OGVs at the multiple monitoring locations the researcher used normalized emission rates. In relation to the total PM2.5 Agrawal et al. found that primary PM2.5 contributions from OGVs range from 8.8% at the monitoring location closest to the port to 1.4% monitored 80 km inland [22]. The scientists concluded that the results of their analysis will be useful in determining the impacts of primary particulate emissions from OGVs upon worldwide communities downwind of port operations.

In the same region of Southern California, Ault et al. studied the impact of emissions during regional shipping transport events [23]. Atmospheric aerosol measurements during time periods with regional transport showed an increase in 0.5-1 μm sized single particles with unique signatures including soot, metals (i.e., vanadium, iron, and nickel), sulfate, and nitrate in La Jolla, California [23]. Ault et al. assumed as well that these particles can be attributed to primary emissions from residual oil sources such as ship engine exhaust. However, the authors of this study state that these particles could also be attributed to emissions of refineries and traffic in the port region as well as to secondary processing during the transport. The results of these measurements showed 2-4 times higher PM2.5 concentrations than typical average concentrations from local sources [23]. Ault et al. therefore conclude that unless significant regulations are imposed these emission sources will become even more important to California air quality as cars and truck emissions. Elevation of PM2.5 concentration in coastal regions due to ship engine emissions was as well reported by a Canadian study carried out by Poplawski et al. They investigated the association between community level concentrations of PM2.5 with cruise ships traffic in Victoria, British Columbia [24]. They obtained data from 2005 to 2008 at a close air quality network site (3.5 km from the study area) and took continuous measurements in the James Bay community over a three-month period during the 2009 cruise ship season. Next to PM2.5 they also investigated concentrations of some gaseous air pollutants. The measurements downwind the port showed an elevation of PM2.5 concentration on weekends when cruise ship activity reached the weekly maximum [24].

In Turkey, Deniz et al. investigated in 2010 within two adjacent studies the shipping emissions in coastal regions with heavy shipping traffic. The first study was carried out in the Candarli Gulf and the second study at the Ambarli Port [25,26]. The objective of the studies was to estimate the amount of major atmospheric components emitted from heavy ship engines. Next to some gaseous air pollutants the studies focused mainly on PM with no distinction between different PM-fractions. The first study in the Candarli Gulf PM investigated the annual shipping emissions from 7520 ships during the year of 2007 and the researcher estimated 57.4 tons per year (t/y) for PM [26]. In the second study Deniz et al. calculated the exhaust emissions from ships at the Ambarli Port by utilizing data acquired in 2005. The total emission from ships at this port was estimated with 36 t/y for PM by the scientists [25].

Healy et al. characterized in a 2009 study single particles from in-port ship emissions in the Port of Cork in Ireland [27]. In this study, the size and composition of freshly emitted individual ship exhaust particles was investigated. For the measurement an aerosol time-of-flight mass spectrometer was used (ATOFMS) co-located with a suite of real-time instrumentations at a site in the Port of Cork. Clustering the collected spectra by using the K-means algorithm, they could identify a unique ship exhaust class containing internally mixed elemental and organic carbon, sodium, calcium, iron, vanadium, nickel and sulfate [27]. During the three week measurement period in August 2008 the Healy et al. group could observe over twenty sharp emission events for this particle type. Coincident increases in mass concentrations of sulfate, elemental carbon and PM2.5 were also observed during these events in this study. Furthermore, simultaneous scanning mobility particle sizer (SMPS) measurements were used. The results of this SMPS monitoring showed that the vast majority of freshly emitted ship exhaust particles is present in the ultrafine mode (< 100 nm diameter) [27]. A second particle class constituted of internally mixed organic carbon, elemental carbon, ammonium and sulfate. The scientists tentatively attributed this second particle class to aged or regionally transported ship exhaust. On basis of these findings, Healy et al. suggested that ATOFMS single particle mass spectra may be useful in determining the contribution of local shipping traffic to air quality in port cities, when used in conjunction with other air quality monitoring instrumentation [27].

## Effect of ship type and fuel type on the PM concentration in ship emissions

In a recent article Johnson et al. investigated size-resolved emission factors for particle number (EF (PN)) and mass (EF (PM)) for 734 individual ship passages. This study was carried out in Sweden near the entrance to the port of Gothenburg [28]. In their experiments an extractive sampling method of the passing ship plumes was used and next to gaseous emissions (CO_2_) the particle number/mass were measured with high time resolution (1 Hz). The place of measurement was situated in an emission control area (ECA) and near to populated areas. The investigation resulted in an average EF (PN) and EF (PM) of 2.55 +/- 0.11 × 10^16 ^(kg fuel) ^-1 ^and 2050 +/- 110 mg (kg fuel)^-1^, respectively [28]. For ships with multiple passages, a great reproducibility for the determined EF was shown and the EF(PN) peaked at particle sizes similar to 35 nm [28]. Interestingly, compared to ships with diesel engines, ships equipped with gas turbine showed smaller particle sizes and less mass. On average 36 to 46% of the emitted particles by number were non-volatile [28].

In a 2011, a real-time study by Jayaram et al. assessed gaseous emissions and PM2.5 emissions of a ship engine powered with different fuel types [29]. The authors measured regulated and unregulated emissions from an in-use marine propulsion engine (EPA Tier 2) on a ferry in a real-time fashion. For comparison with the certification standards and across biodiesel blends the investigated engine was operated following the loads in ISO 8178-4 E3 cycle. Furthermore, real-time measurements were made during a typical cruise in the bay. As a result Jayaram et al. were able to demonstrate that in-use PM2.5 emission factors were within the not-to-exceed standard for Tier 2 marine engines. The comparison of the fuels showed for PM2.5 a reduction of 16% on ships fuelled with Biodiesel B20 and of 25% on ships fuelled with Biodiesel B50 [29]. Furthermore, reductions in the volume mean diameter and total number concentration in the accumulation mode with increasing biodiesel blends was observed. These findings were consistent with trends found in gravimetric PM2.5 mass emissions [29]. In other studies similar trends of particle size and number reduction in accumulation mode with biodiesel was reported [30,31]. Jayaram et al. were able to demonstrate the important effects of ocean/bay currents on emissions. Due to this effect, PM2.5 mass increased about 6-fold and ultrafine particles (UFP) disappeared [29]. Based on the findings, the authors conclude that for the development of emission inventories the effect of ocean currents should be considered. In this respect, in-use measurements may provide necessary data for accurate inventories [29].

In regard to an approaching fuel switch within the marine sector, a study was conducted by Winnes et al. with the aim to investigate the effects of different fuel types on ship exhaust gas composition and emission factors with a focus on particles [32]. The field emission measurements were carried out on the 4500-kW four-stroke main engine on-board a product tanker. In the study, heavy fuel oil and marine gas oil were tested on the same engine for comparable load settings [32]. In this study PM with no distinction of different fraction was measured. The authors reported for heavy fuel oil generally higher specific PM emissions than for marine gas oil but for the smallest size-fraction containing particles 0.30- 0.40 μm in diameter, the opposite was observed [32]. The authors' conclusion of these findings is that further regulation is needed to reduce small-sized particles and hence negative health effects of particles from ships.

## Characterization of the PM composition in ship emissions

Only few published studies focused on the characterization of the PM composition in ship emissions. One project was an international study carried out by Moldanova et al. (2009) that investigated PM in emissions from a large ship diesel engine [33]. The engine was fueled with heavy fuel oil (HFO). The investigation of the emitted PM included mass, size distribution, chemical composition and microphysical structure of the PM. The study assessed an emission factor for PM of 5.3 g (kg fuel) ^-1^. Further, the mass size distribution showed increased particles of the accumulation mode (mean diameter of 0.5 μm) and the coarse mode (around 7 μm) [33]. The investigation demonstrated a domination of organic carbon (OC), ash and sulfate in the PM composition whereas elemental carbon (EC) composed only a few percent. It is to point out that increase of PM in the exhaust upon cooling, was associated with increase of OC and sulfate. Interestingly, cooling of the exhaust as well showed an effect on the quantity of polycyclic aromatic hydrocarbons (PAHs) absorbed on the surface of particulates. Whereas the analysis of the adsorbed phase in the cooled exhaust presented a rich mixture of PAH species with molecular mass between 178 and 300 atomic mass units (amu) the hot exhaust showed only 4 amu of PAH [33]. In the performed microstructure and elemental analysis of ship combustion residuals three following distinct morphological structures with different chemical composition were found: soot aggregates, significantly metal polluted; char particles, clean or containing minerals; mineral and/or ash particles [33]. In addition, the researcher could observe organic carbon particles of unburned fuel or/and lubricating oil origin were. It should be pointed out that hazardous constituents from the combustion of heavy fuel oil (V, Ni, Ca, and Fe (iron)) were observed in the PM samples as well. These metals were also used in other studies as marker for ship emissions as described above.

In a 2009 study carried out by Murphy et al. the particulate exhaust from a modern container ship burning heavy fuel oil was characterized [34]. The ship emissions were measured shipboard and airborne with a focus on the chemical composition and water-uptake behavior of particulate matter in the exhaust. The following results were obtained: The mass ratio of particulate organic carbon to sulfate was 0.23 +/- 0.03 at the base of the ship stack and 0.30 +/- 0.01 in the airborne exhaust plume [34]. The additional organic mass in the airborne plume was concentrated largely in particles below 100 nm in diameter [34]. The organic to sulfate mass ratio in the exhaust aerosol remained constant during the first hour of plume dilution into the marine boundary layer [34]. The authors also reported that the mass spectrum of the organic fraction of the exhaust aerosol appeared to be predominantly hydrocarbon-like organic (HOA) material [34]. The background aerosol contained a lower organic mass fraction than the fresh plume with much more oxidized organic component [34]. With a conducted analysis of the water-uptake behavior of particulate in the exhaust the researcher showed that a volume-weighted mixing rule is able to accurately predict hygroscopic growth factors in the background aerosol. However, measured and calculated growth factors do not agree for aerosols in the ship exhaust plume. The researchers estimated the particle number emission factor at 1.3 × 10^16 ^(kg fuel) ^-1^, with less than 1/10 of the particles having diameters above 100 nm and 24% of particles (> 10 nm in diameter) activate into cloud droplets at 0.3% supersaturation [34].

A third study that aimed to characterize the PM composition in ship emissions focused on the urban area of Venice in Italy. Contini et al. estimated in this study the direct influence of ship traffic on atmospheric PM2.5 and PM10. Next to PM, the study also estimated fifteen PAHs. The sample collection was performed over the summer when ship traffic is expected to be at a maximum and on three sites in that area [35]. From the received results the researcher concluded that the PM daily concentrations are not sufficiently detailed for the evaluation. Therefore they developed a new methodology, based on high temporal resolution measurements coupled with wind direction information and the database of ship passages of the Harbor Authority of Venice [35]. PM10 and PM2.5 were monitored with optical detectors operating at a high temporal resolution [35]. With this new setup, the study showed that direct contribution of ships traffic to PM_2.5 _and to PM_10 _ranges from 1% up to 8% [35].

## Conclusion

**Figure 2:**
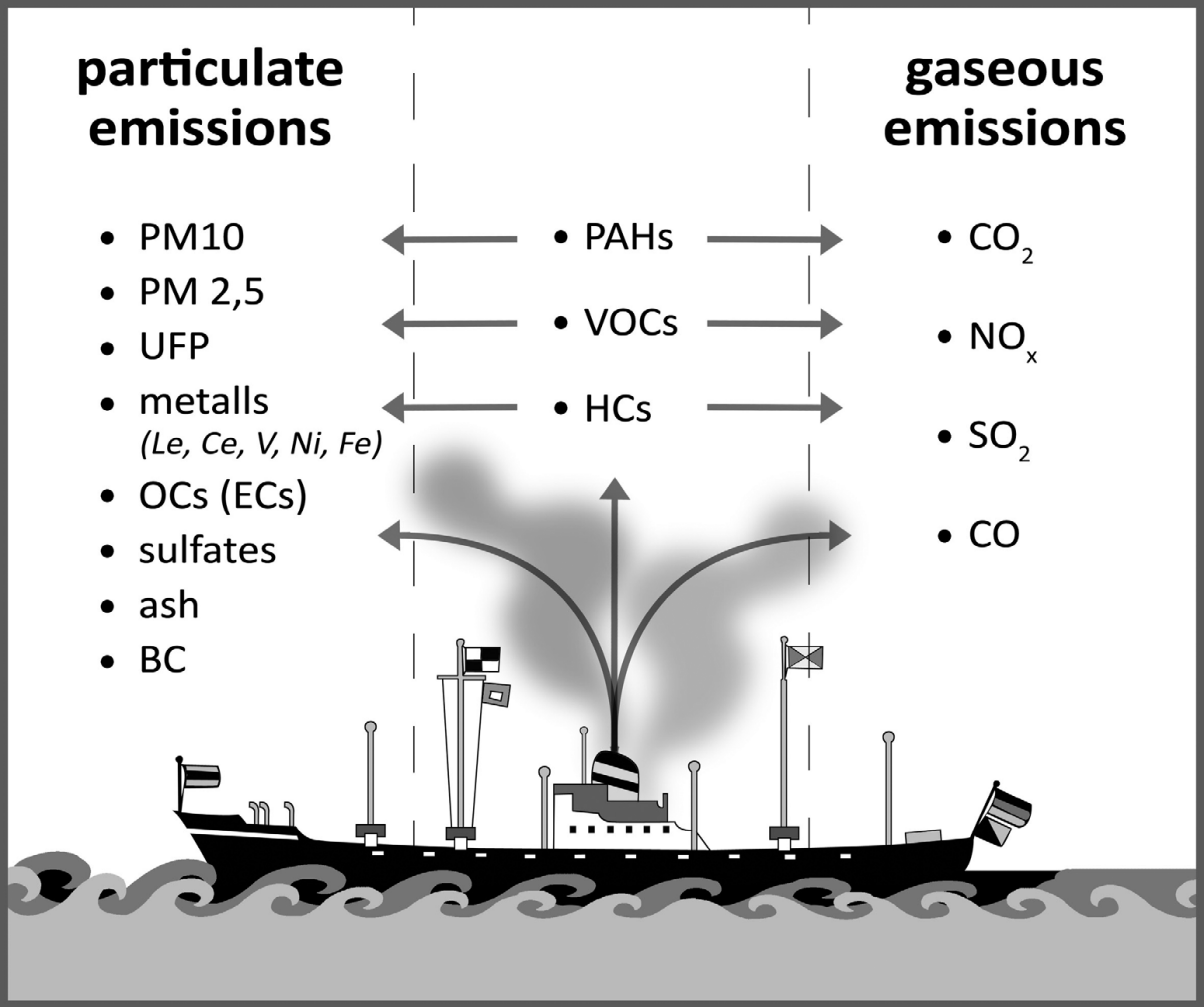
**Characterized compounds of emissions from marine vessel engines as reported in the reviewed articles**.

This review of the recent published literature shows that port and populated coastal areas with heavy ship traffic are affected by exhausts of particulates from marine vessels. From the different studies it can be concluded that further international regulations are necessary to assess vessel-related air pollution due to ship traffic emissions. In this respect, recent articles have shown that simple fuel change of marine vessel engines alone may not sufficiently reduce effects caused by ship emissions. The few publications that analyzed the composition of PM in ship emissions show the importance of this kind of approach. Knowledge about the nature of the compounds of ship emissions helps to discriminate these pollutant sources from others in the port areas and coastal regions (Figure 1). Promising markers for ship engine exhaust are specific metals like vanadium and nickel although there are is a large variety and quantity of other harmful substances present (Figure 2). Since there is still only little published data regarding the quality and quantity of particles emitted from ship engines, modern scientometric tools which are in use for the analysis of other research areas are not applicable in this area [36-44].

## Competing interests

The authors declare that they have no competing interests.

## Authors' contributions

DM, SU, MT, DK, DAG have made substantial contributions to the conception and design of the review, acquisition of the review data and have been involved in drafting and revising the manuscript. All authors have read and approved the final manuscript.
